# Letter from Dr. W. T. Taylor, of Kentucky

**Published:** 1873-08

**Authors:** 


*Editors Southern Medical Record:*

The July number of the RECORD is just received, its pages freighted, as usual, with good things.

The high "ethical" grounds taken by the RECORD can not be too highly commended, and if its policy in this respect were adopted by other medical journals, the profession would be, by all odds, the gainer. Medical ethics, it seems to me, deserve much more attention now than ever before, and treatises on this subject ought to be widely circulated and carefully studied. Formerly, the length of time to which medical pupilage extended, the solid elementary instruction which was insisted upon, the distinction which the diploma conferred, and various other causes, combined to make the young practitioner conscious of his dignity, and sedulous to preserve his honor unsullied. But, alas! from what a height have we fallen! When we look around us, how many schools do we see, where the teachers are totally unqualified for the work they have assumed—the pupils raw, the instruction scanty, and the diploma a false certificate? Can it be wondered at, that the medical profession is infested with a horde of persons who have no other conception of it than as a trade, and who are entirely ignorant of those precepts and principles which have become a part of the moral nature of all who have received a liberal education? We need not only counsel to guide us in our intercourse with our patients and professional brethren, that the heart-burnings, jealousies and disappointments may be prevented, which so often beset the practitioner's path, but also that we may learn how to appreciate the false systems which tempt the unwary to barter their inheritance of scientific wealth for momentary applause, and to cherish for the noblest and most humane of pursuits a reverent love that never fails to make one honored and esteemed, and that secures for him the priceless blessing of a conscience at peace with itself. It should be the object of every medical journal to elevate the profession—and, in order to do this, every thing which tends, directly or indirectly, in an opposite direction, should be sedulously excluded. When we look over the field, we are painfully aware of how much the profession needs improvement, and the difficulties that beset us right at the outset. How difficult, may be surmised, when we notice how few, comparatively, of our eminent physicians are willing to insist upon a thorough, liberal education as a preliminary to medical studies, or to denounce the system of medical training which a large proportion, at least, of the schools furnish as being what it really is : a system for degrading the profession still lower, and imposing upon the community physicians who possess scarcely the first rudiments of a medical knowledge. How little encouragement is held out to such as, from love of knowledge, or desire of distinction, endeavor to obtain a thorough education. How strong a temptation for them to abate their diligence, and cast in their lot with the mass who find, by a much easier path, both reputation and a competency. It is not a rare thing to see the half-educated or the wholly ignorant man, who has boldness enough to make high pretensions, and adroitness enough to palm off natural effects as the result of his skill alone, inspire more confidence than the conscientious and, therefore, circumspect physician. His very ignorance is a charm. He is supposed, by the intuitions of his genius, to discover more than the learned practitioner can do by study and observation ; not a few, even, of the genteel vulgar imagine that he has a "gift" which makes him quite independent of the ordinary processes of acquiring skill. Unfortunately, this error is fostered by an equally gross one in the profession itself. There are so many physicians of little talent and less learning, and who openly affect to disdain both, as compared with tact and experience, that we ought not to wonder at the large number beyond our limits who despise what our own brethren undervalue. How large a number, too, of those who annually enter the profession, whose general and literary education is absolutely null, and who would have made Doctors of Medicine after a period, we will not say of study, but of attendance upon lectures, which is only sufficient to reveal the impossibility of their ever being fit to treat disease. So long as a large proportion of medical men are wholly uneducated, or only instructed enough to make them dangerous to the sick, we have no right to complain that the public prefers employing professed charlatans, rather than those who, with the title of Doctor, have none of the accomplishments which belong to so honorable a name. It is to our disgrace, as a body, that we do not insist upon a higher standard of professional and general education than is now exacted. It can not be expected that men who have never formed habits of study in early life, who know little or nothing of the art of observation or the science of reasoning, should become really accomplished physicians.

To bring the profession and the public to a sense of this error, it is necessary that they should have special instruction in this direction, and the medical journal is preëminently the instrumentality by which this knowledge is to be made available.

The field of "medical ethics" is a wide one, and, notwithstanding the great number of medical journals in this country, it is comparatively uncultivated. We are glad to see that the RECORD does not ignore this subject. There seems to be a notion, even among some physicians, that "medical etiquette" is some cumbrous ceremonial, which clogs the freedom of the physician's actions, and ought, therefore, to be laid aside whenever the case in hand is really grave. This shows, among other things, how utterly misapprehended are the nature and objects of the medical profession. Medical etiquette is only a form of common honesty, and it were quite as just to bid one forget that he is an honest man when he is tempted to do wrong, as to beg the physician to forget those rules which have been framed to guide him in the intricate paths, and amid the temptations of his professional life. All moral law is favorable to freedom of action in the good; it is a stumbling-block to the bad alone. So it is eminently true, in this case, that a strict adherence to ethical rules "favors freedom of intercourse, by maintaining mutual confidence; while a disregard of them destroys the freedom, by engendering mutual distrust."

But I am trespassing too much upon your time and space, and will close, with the fervent wish, that the RECORD may go on growing in usefulness, as it has in the good graces of the profession, until our noble calling be free from the glaring errors and inconveniences which have so long clogged its progress.

With much respect, I am, gentlemen, as ever, truly yours,

W. T. TAYLOR, M.D.

